# Endoscopic diagnosis of gastric and oesophageal cancer in Lusaka, Zambia: a retrospective analysis

**DOI:** 10.1186/s12876-024-03187-x

**Published:** 2024-04-01

**Authors:** Violet Kayamba, Malambo Mubbunu, Paul Kelly

**Affiliations:** 1 Tropical Gastroenterology and Nutrition Group, University of Zambia School of Medicine, Nationalist Road, PO Box 50398, Lusaka, Zambia; 2 Blizard Institute, Barts & The London School of Medicine and Dentistry, Queen Mary University of London, 4 Newark Street, E1 2AT, London, UK

**Keywords:** Gastric cancer, Oesophageal cancer, Duodenal ulcer, Peptic ulcer, Endoscopy, Audit

## Abstract

**Introduction:**

There are uncertainties surrounding the spectrum of upper gastrointestinal (UGI) diseases in sub-Saharan Africa. This is mainly due to the limitations of data collection and recording. We previously reported an audit of UGI endoscopic diagnoses in Zambia spanning from 1977 to 2014. We now have extended this analysis to include subsequent years, in order to provide a more comprehensive picture of how the diagnoses have evolved over 4 decades.

**Methods:**

We combined data collected from the endoscopy unit at the University Teaching Hospital (UTH) in Lusaka during a previous review with that collected from the beginning of 2015 to the end of 2021. Since 2015, an electronic data base of endoscopy reports at the UTH was kept. The electronic data base was composed of drop-down menus that allowed for standardised reporting of findings. Collected data were coded by two experienced endoscopists and analysed.

**Results:**

In total, the analysis included 25,849 endoscopic records covering 43 years. The number of endoscopic procedures performed per year increased drastically in 2010. With the exception of the last 2 years, the proportion of normal endoscopies also increased during the time under review. In total, the number of gastric cancer (GC) cases was 658 (3%) while that of oesophageal cancer (OC) was 1168 (5%). The number of GC and OC diagnoses increased significantly over the period under review, *(p* < 0.001 for both). For OC the increase remained significant when analysed as a percentage of all procedures performed (*p* < 0.001). Gastric ulcers (GU) were diagnosed in 2095 (8%) cases, duodenal ulcers (DU) in 2276 (9%) cases and 239 (1%) had both ulcer types. DU diagnosis showed a significantly decreasing trend over each decade (*p* < 0.001) while GU followed an increasing trend *(p* < 0.001).

**Conclusions:**

UGI endoscopic findings in Lusaka, Zambia, have evolved over the past four decades with a significant increase of OC and GU diagnoses. Reasons for these observations are yet to be established.

## Introduction

There is a paucity of information on the profile of upper gastrointestinal (UGI) diseases in sub-Saharan Africa (SSA), but a recent increase in related publications has shed light on some predominant conditions. Globally, there is evidence of decreasing incidence of gastric cancer (GC) [1] and peptic ulceration [2], but with a paradoxical increase of oesophageal cancer (OC) in some regions, especially adenocarcinoma [3]. Due to limited data from SSA, accurate time trends have been difficult to ascertain. Endoscopic diagnosis of UGI diseases is one avenue through which these conditions can be reliably described, though largely restricted to hospital patients.

We previously reported results for an audit of endoscopy records at the University Teaching Hospital (UTH) including both upper and lower GI diagnoses [4, 5]. Endoscopy services in Zambia began in 1977, with the first public services being offered at UTH in Lusaka, Zambia. UTH is a public institution and is one of the largest referral hospitals in Zambia, attending to patients from all 10 provinces of the country. Endoscopy services at UTH have over the years been fairly consistent, with occasional breaks when there was no functioning endoscopic equipment or trained endoscopists. Generally, a well-equipped unit would have a good proportion of upper gastroscopes, colonoscopes, duodenoscopes and processors [6] and the UTH unit has for most of the years been able largely to sustain this requirement.

In our previous audit of UGI endoscopies, we described diagnostic trends, highlighting changes that were influenced by the onset, and subsequent control of the Human Immunodeficiency Virus (HIV) pandemic [4]. Prior to the introduction of anti-retroviral treatment (ART), opportunistic infections dominated GI related research work in SSA. This resulted in a skewed view of the spectrum of GI diseases. We previously reported that HIV had no association with GC [7] or *Helicobacter pylori (H. pylori)* infection [8], but that it was linked to a high occurrence of hypochlorhydria [9, 10]. Additional evidence showed that viral suppression with ART did not reverse HIV associated hypochlorhydria [11]. Our previous endoscopy audit showed a significant reduction of HIV related UGI diagnoses, Kaposi Sarcoma and oesophageal candidiasis since ART became widely available [4]. However, no such reductions were noted for UGI malignancies or benign ulceration, which are the main focus of our current report.

We have now aggregated all the endoscopy reports since the inception of endoscopy services at UTH in 1977 to the end of 2021, in order to evaluate trends of gastric and oesophageal cancer diagnosis and peptic ulceration. The University of Zambia Biomedical Ethics Committee granted an ethics review waiver for this publication.

## Methods

### Retrieval of records

We previously retrieved paper records of endoscopy reports from 1977 to 2014 and transcribed them into an electronic data base for analysis. Since 2015, the UTH endoscopy unit developed a bespoken structured electronic recording system with drop down menus of the most common conditions that enabled uniform reporting, using previous written records as a guide. The electronic records included patient details, with endoscopic findings recorded for each site; oesophagus, stomach and duodenum.

For this audit, we evaluated reports on GC, OC, gastric and duodenal ulcers. For OC, information on tumour location (measured as distance from the incisors in centimetres) and patency of the lumen was reported. For these measurements, we considered a distance less than 25 cm as being in the upper, 25 to 30 cm middle, 31 to 40 cm lower oesophagus. Those distal to 40 cm were deemed to be at the gastroesophageal junction. Inability of the 9.8 mm diameter endoscope to pass beyond the tumour was reported as luminal occlusion. For GC, tumour location was either on the gastro-oesophageal junction, fundus, corpus or antrum. However, some tumours were seen to affected more than one gastric site and that information was recorded as such. We also collected information on peptic ulcers; gastric and duodenal lesions. Data were aggregated with the previous dataset, forming a combined set on which we are currently reporting.

As a significant number of records did not have patient demographics, our report does not include demographic characteristics.

### Data analysis

On the endoscopy electronic data base, we analysed all reports that had a diagnosis written as “oesophageal cancer”, “gastric cancer”, “gastric ulcer” or “duodenal ulcer”. However, there were a few overlaps in these diagnoses, with some patients having more than one of these. The data were coded and exported to StataCorp. 2017. *Stata Statistical Software: Release 15*. College Station, TX: StataCorp LLC, for analysis. To create the graphs, we used GraphPad Prism version 9.0, GraphPad Software, San Diego, California USA. Time trends were analysed by year of diagnosis. We reported proportions using percentages. Significance testing was carried out using the nptrend command for non-parametric test of trend for the ranks of across ordered groups. We also grouped the years by decade and analysed using χ^2^test appropriate. For determination of significance, we used a probability value of less than 0.05.

## Results

**Fig. 1:**
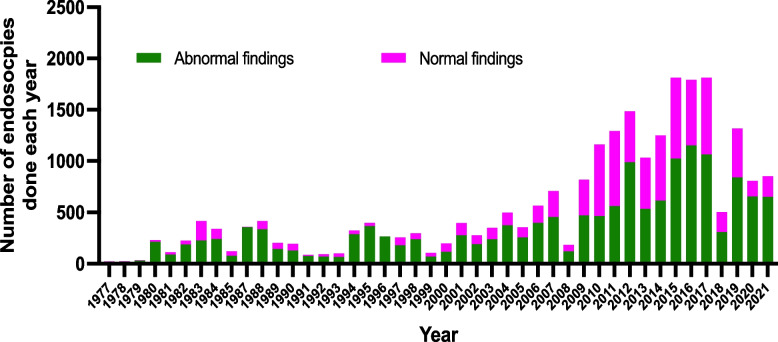
Total number of endoscopies performed each year, stratified by normal or abnormal findings

We analysed records of 25, 849 endoscopies from January 1st 1977 to December 31st 2021. Categorised by 10-year bands; 1674 (6%) of the procedure were done between 1977 and 1986; 2627 (11%) between 1987 and 1996; 3388 (13%) between 1997 and 2006; 12,868 (50%) between 2007 and 2016 and 5292 (20%) between 2017 and 2021. Overall, 8662 (36%) of the endoscopies had no visible mucosal lesions in either the oesophagus, stomach or duodenum. The proportion of normal endoscopies was significantly higher in later years, falling off again in 2020 and 2021, *p* < 0.001. The highest proportion of normal endoscopies was between 2009 and 2017, with the highest recording in 2010 at 60%, Fig. 1.

### Gastric and oesophageal cancer diagnosis

During the period under review, a total of 685 and 1168 cases of gastric and oesophageal cancer were diagnosed respectively. Of the GC cases, we had data on exact tumour location for 117 cases, of which the majority (71%) were located in distal parts of the stomach (Table 1). Thirty of the cases were located at the gastroesophageal junction. We had data on the location of OC for 333 cases, of which 69 (22%) were in the upper, 138 (44%) middle and 104 (34%) in the lower oesophagus respectively (Table 1). Luminal occlusion was significantly higher in tumour located in the upper oesophagus than the lower parts, *p* < 0.001.

**Table 1:** Anatomical location of gastric and oesophageal tumours as reported by the endoscopists

Gastric cancer	Number (%)			
Fundus	18 (17)			
Body	39 (36)			
Antrum	38 (35)			
More than one site	14 (12)			
Oesophageal cancer	Number (%)	Luminal occlusion, *n* (%)	No occlusion, *n* (%)	Missing data on occlusion, *n* (%)
Upper oesophagus	69 (22)	39 (57)	27 (39)	3 (4)
Middle oesophagus	138 (44)	71 (51)	65 (47)	2 (1)
Lower oesophagus	104 (34)	32 (31)	70 (67)	2 (2)
*p*-value computed using Chi^2^	< 0.001			
Gastroesophageal junction	Number	Luminal occlusion	No occlusion	
	30	4 (13)	17 (57)	9 (30)

### Time trend diagnosis of gastric and oesophageal cancer

**Fig. 2:**
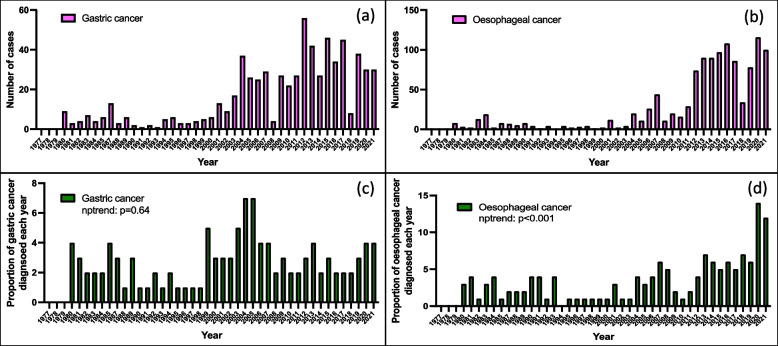
Endoscopic diagnosis of gastric and oesophageal cancer by year; (**a**) gastric cancer total numbers, (**b**) oesophageal cancer total numbers, (**c**) gastric cancer as a proportion of all endoscopies performed that year and (**d**) oesophageal cancer as a proportion of all endoscopies performed that year

The numbers of gastric and oesophageal cancer cases diagnosed in the UTH endoscopy unit increased steadily over four decades. Analysis of these diagnoses as a proportion of all endoscopies, showed a statistically significant increase for both cancers when categorised by decade, *p* < 0.001. However, analysis of the trend by uncategorized years, showed significant increase for oesophageal cancer, *p* < 0.001, but not gastric cancer *p* = 0.64 (Fig. 2).

### Time trend diagnosis of gastric and duodenal ulcers

**Fig. 3:**
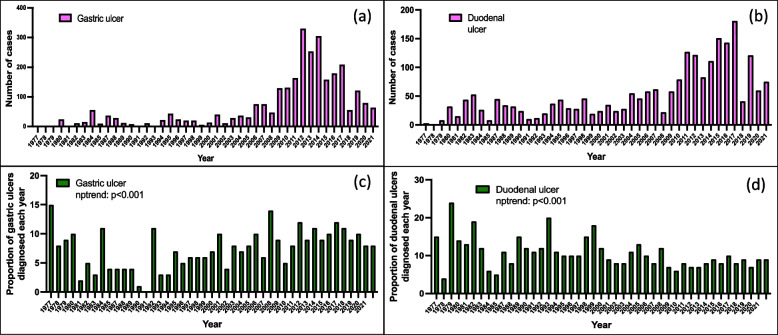
Endoscopic diagnosis of gastric and duodenal ulcers by year; (**a**) gastric ulcers total numbers, (**b**) duodenal ulcers total numbers, (**c**) gastric ulcers as a proportion of all endoscopies performed that year and (**d**) duodenal ulcers as a proportion of all endoscopies performed that year

A total of 4610 (18%) cases of peptic ulcers were diagnosed, and of these, 2095 (46%) were gastric ulcers, 2276 (49%) duodenal ulcers and 239 (5%) had both. The numbers of peptic ulcer diagnosis increased steadily over the period under review (Fig. 3). Analysis of these diagnoses as a proportion of all endoscopies, showed a significant decrease in diagnosis of duodenal ulcers (*p* < 0.001), but a significant increase in the diagnosis of gastric ulcers (*p* < 0.001; Fig. 3). Similarly, analysis by decade showed that diagnosis of duodenal ulcers decreased from 11% in the first decade to 9% in the last (*p* < 0.001) while gastric ulcer diagnoses increased from 7% in the first decade to 10% in the last, *p* < 0.001, Table 2.

**Table 2:** Proportion of gastric and duodenal ulcers diagnosed per decade since 1977

Decade	Gastric ulcers	Duodenal ulcers
1977–1986	111/ 1658 (7%)	190/ 1668 (11%)
1987–1996	113/ 2548 (4%)	287/ 2592 (11%)
1996–2006	254/ 3360 (8%)	363/ 3365 (10%)
2007–2016	1088/ 12,185 (9%)	958/ 12,618 (8%)
2017–2021	529/ 5292 (10%)	478/ 5292 (9%)
*p*-value determined using Chi^2^	< 0.001	< 0.001

## Discussion

We conducted an audit of endoscopy records covering over four decades. Our major findings were of a significant increase in the total number of GC and OC diagnoses over 43 years. For OC the increase remained significant when analysed as a percentage of all procedures performed. In addition, the diagnosis of duodenal ulceration decreased while that of gastric ulcers increased during the period under study.

The occurrence of GC and OC is variable throughout a world. A recently published analysis reviewed that GC:OC ratio in men exceeded 10 in several South American countries with further evidence that the relationship between GC and OC incidence differed by sub-type [12]. Li et al., reported that generally, non-cardia GC was negatively correlated with adenocarcinoma of the oesophagus, but positively correlated with squamous cell carcinoma [12]. Relative ratios for most of sub-Saharan Africa (SSA) are difficult to determine accurately due to limited data [13] and low cancer research output [14]. Endoscopic services in SSA are limited and in many cases only serve the elite few [15]. This is due to the high cost of establishing and maintaining these units. Several strategies have been proposed aimed at improving endoscopy services in hospitals to make them more cost-effective [16]. In many centres, dyspeptic symptoms are a major indication for endoscopy but if there are no other signs or symptoms, the diagnostic yield is low [17–19]. A study from the Netherlands revealed that patient’s education on gastric function, dyspepsia and upper GI endoscopy decreased the need for procedures to be done among uninvestigated individuals with dyspepsia [20]. It is obvious that there is need to streamline requests for endoscopy to make them more appropriate, limiting the wastage of resources [21]. For example, using GI alarm symptoms, such as haematemesis, melaena, unexplained weight loss, dysphagia, persistent vomiting or microcytic anaemia results in better endoscopic yield, although there is evidence that their positive predictive value for malignancy is also low [22]. The negative predictive value of an absence of these alarm symptoms tends to be much better at predicting GI malignancy. International guidelines, advise that endoscopies be done in all dyspeptic patients above the age of 50 years, even in the absence of alarm symptoms [23]. The validity of these guidelines is however not certain in settings such as Zambia, where close to a quarter of GC cases are below the age of 45 years [7].

Basic demographic characteristics or risk factors on their own do not adequately distinguish structural from functional diseases in patients with upper GI symptoms [24]. A study from Eldoret in Kenya showed that about a third of the endoscopies were normal, which was similar to Ethiopia at 28% [18, 25]. In Nigeria, the proportion on normal endoscopies was 26% [26], suggesting that our 43-year average of 36% was quite high. With the realisation that the number of normal endoscopies being done in our low resource unit was too high, we recently introduced a screening system, in which all requests have to be scrutinised by one of the unit doctors before booking for endoscopy. This might explain the reduction of normal endoscopies for 2020 and 2021. In addition, during 2020 and 2021, unit closures were instituted in many hospitals including the UTH due to the coronavirus disease 19 (COVID 19) pandemic. During these shut downs, we could only perform emergency procedures for patients with haematemesis or dysphagia. Endoscopic requests for procedures that were not deemed urgent were not honoured. Such patients were provided with alternative approaches to therapy while other went to private centres for endoscopy. This could also explain the reduced numbers of normal endoscopies during that period. Similar disruptions to endoscopic services were also reported elsewhere [27].

The number of both GC and OC diagnosis increased over the past four decades at UTH. However, when we evaluated the numbers in relation to total numbers of endoscopies done each year, the increase was significant for OC but not GC. This should be interpreted cautiously as endoscopy records might not accurately represent what is pertaining at national level. The overall ratio of OC to GC was 2 to 1, a proportion similar to that reported in Uganda [28]. Time trends for GC in SSA are very limited. This audit is now suggesting that similar to other parts of the world [1], the incidence of GC in SSA is also decreasing. *H. pylori* is the main risk factor for GC and its prevalence is very high in SSA. With advanced treatment approached and improved socio-economic environments, it is possible that the prevalence of *H. pylori* is also decreasing in SSA with an effect on the numbers of GC. The increase in the proportion of OC cases being diagnosed is however striking. More research needs to be conducted in order to understand the drivers of this increase. We previously reported that biomass smoke was a significant risk factor for both GC and OC but mechanisms involved in this association are yet to be established [29, 30].

We also evaluated the anatomical location of the cancers. OC was predominantly located in the upper and middle areas with significantly less occlusion with the distal tumours. We did not have histology reports, and therefore cannot make reference to the sub-types. However, we know from past work that the predominant type of OC in Zambia is squamous cell carcinoma [31]. Globally, the occurrence of non-cardia or distal gastric cancer is decreasing, with a relative increase of cardia tumours [32]. Generally, *H. pylori* infection tends to predispose to non-cardia GC. Similar to other SSA countries [33], we found that most of the tumours were non-cardia, suggesting that they could be driven by *H. pylori* infection, but also that unique risk factors such as biomass smoke exposure could be influencing GC development in the population [30].

The proportion of GU diagnosis increased significantly over that past four decades, while that of DU decreased. *H. pylori* infection is a significant risk factor for both ulcer types, but GU tend to have a wider range of other aetiological factors as well. Therefore, this differential occurrence could be due to improved *H. pylori* treatments, affecting DU more than GU. Over the whole period under review, the prevalence of GU was 8% while that of DU was 9%. These proportions are not dissimilar to that reported by a Cuban study at 6.2% [34].

Analysis of temporal trends was limited by the changing number of endoscopies done each year, resulting in varying denominators. We did not have information on the exact dates when equipment was being upgraded or changed, and therefore this is a limitation of our audit. Our audit was also limited by infrequently recorded basic characteristics of the cases we evaluated. It would have provided an indication of sex differences. We did not have data for 1986. Another limitation was in the inconsistent documentation of exact anatomical location of the cancer. This resulted in a limited number of cases analysed for this purpose.

## Conclusion

The occurrence of OC and GU is increasing in the Zambian population, while that of DU is decreasing. No such changes are evident for GC.

## Data Availability

The datasets used and/or analysed during the current study are available from the corresponding author on reasonable request.

## References

[1] Morgan E, Arnold M, Camargo MC, Gini A, Kunzmann AT, Matsuda T, Meheus F, Verhoeven RHA, Vignat J, Laversanne M, Ferlay J, Soerjomataram I (2022). The current and future incidence and mortality of gastric cancer in 185 countries, 2020-40: a population-based modelling study. EClinicalMedicine..

[2] Wang AY, Peura DA (2011). The prevalence and incidence of helicobacter pylori-associated peptic ulcer disease and upper gastrointestinal bleeding throughout the world. Gastrointest Endosc Clin N Am.

[3] Huang J, Koulaouzidis A, Marlicz W, Lok V, Chu C, Ngai CH, Zhang L, Chen P, Wang S, Yuan J, Lao XQ, Tse SLA, Xu W, Zheng ZJ, Xie SH, Wong MCS (2021). Global burden, risk factors, and trends of esophageal Cancer: an analysis of Cancer registries from 48 countries. Cancers (Basel).

[4] Kayamba V, Sinkala E, Mwanamakondo S, Soko R, Kawimbe B, Amadi B, Zulu I, Nzaisenga JB, Banda T, Mumbwe C, Phiri E, Munkonge P, Kelly P (2015). Trends in upper gastrointestinal diagnosis over four decades in Lusaka, Zambia: a retrospective analysis of endoscopic findings. BMC Gastroenterol.

[5] Kayamba V, Nicholls K, Morgan C, Kelly P (2018). A seven-year retrospective review of colonoscopy records from a single Centre in Zambia. Malawi Med J.

[6] Axon ATR (2020). Fifty years of digestive endoscopy: successes, setbacks, solutions and the future. Dig Endosc.

[7] Kayamba V, Asombang AW, Mudenda V, Lisulo MM, Sinkala E, Mwanamakondo S, Mweemba I, Kelly P (2013). Gastric adenocarcinoma in Zambia: a case-control study of HIV, lifestyle risk factors, and biomarkers of pathogenesis. S Afr Med J.

[8] Kayamba V, Butt J, Varga M, Shibemba A, Piazuelo MB, Wilson KT, Zyambo K, Mwakamui S, Mulenga C, Waterboer T, Epplein M, Heimburger DC, Atadzhanov M, Kelly P (2022). Serum antibodies to *helicobacter pylori* antigens are associated with active gastric inflammation but not gastric cancer in patients seen at the university teaching Hospital in Lusaka, Zambia. Malawi Med J.

[9] Kelly P, Shawa T, Mwanamakondo S, Soko R, Smith G, Barclay GR, Sanderson IR (2010). Gastric and intestinal barrier impairment in tropical enteropathy and HIV: limited impact of micronutrient supplementation during a randomised controlled trial. BMC Gastroenterol.

[10] Hodges P, Kelly P, Kayamba V (2021). Helicobacter pylori infection and hypochlorhydria in Zambian adults and children: a secondary data analysis. PLoS One.

[11] Kayamba V, Shibemba A, Zyambo K, Heimburger DC, Morgan D, Kelly P (2018). HIV related hypochlorhydria does not appear to respond to anti-retroviral therapy in Zambian adults: a case control study. Pan Afr Med J..

[12] Li M, Park JY, Sheikh M, Kayamba V, Rumgay H, Jenab M, Narh CT, Abedi-Ardekani B, Morgan E, de Martel C, McCormack V, Arnold M (2022). Population-based investigation of common and deviating patterns of gastric cancer and oesophageal cancer incidence across populations and time. Gut..

[13] Ngwa W, Addai BW, Adewole I, Ainsworth V, Alaro J, Alatise OI, Ali Z, Anderson BO, Anorlu R, Avery S, Barango P, Bih N, Booth CM, Brawley OW, Dangou JM, Denny L, Dent J, Elmore SNC, Elzawawy A, Gashumba D, Geel J, Graef K, Gupta S, Gueye SM, Hammad N, Hessissen L, Ilbawi AM, Kambugu J, Kozlakidis Z, Manga S, Maree L, Mohammed SI, Msadabwe S, Mutebi M, Nakaganda A, Ndlovu N, Ndoh K, Ndumbalo J, Ngoma M, Ngoma T, Ntizimira C, Rebbeck TR, Renner L, Romanoff A, Rubagumya F, Sayed S, Sud S, Simonds H, Sullivan R, Swanson W, Vanderpuye V, Wiafe B, Kerr D (2022). Cancer in sub-Saharan Africa: a lancet oncology commission. Lancet Oncol.

[14] Kayamba V, Mutale W, Cassell H, Heimburger DC, Shu XO (2021). Systematic review of Cancer research output from Africa, with Zambia as an example. JCO Glob Oncol.

[15] Mwachiro M, Topazian HM, Kayamba V, Mulima G, Ogutu E, Erkie M, Lenga G, Mutie T, Mukhwana E, Desalegn H, Berhe R, Meshesha BR, Kaimila B, Kelly P, Fleischer D, Dawsey SM, Topazian MD (2021). Gastrointestinal endoscopy capacity in eastern Africa. Endosc Int Open.

[16] Sun E, Hughes ML, Enslin S, Bull-Henry K, Kaul V, Littenberg GD (2021). The role of the gastrointestinal hospitalist in optimizing endoscopic operations. Gastrointest Endosc Clin N Am.

[17] Ntola VC, Pillay TG, Ramklass S, Sibanda W (2019). An audit of upper gastrointestinal endoscopy performed on patients at prince Mshiyeni memorial Hospital in Durban, KwaZulu-Natal. S Afr J Surg.

[18] Ayuo PO, Some FF, Kiplagat J (2014). Upper gastrointestinal endoscopy findings in patients referred with upper gastrointestinal symptoms in Eldoret, Kenya: a retrospective review. East Afr Med J.

[19] de Jong JJ, Lantinga MA, Drenth JP (2019). Prevention of overuse: a view on upper gastrointestinal endoscopy. World J Gastroenterol.

[20] de Jong JJ, Lantinga MA, Tan ACITL, Aquarius M, Scheffer RCH, Uil JJ, de Reuver PR, Keszthelyi D, Westert GP, Masclee AAM, Drenth JPH (2021). Web-based educational intervention for patients with uninvestigated dyspepsia referred for upper gastrointestinal tract endoscopy: a randomized clinical trial. JAMA. Intern Med.

[21] Dakubo JC, Clegg-Lamptey JN, Sowah P (2011). Appropriateness of referrals for upper gastrointestinal endoscopy. West Afr J Med.

[22] Vakil N, Moayyedi P, Fennerty MB, Talley NJ (2006). Limited value of alarm features in the diagnosis of upper gastrointestinal malignancy: systematic review and meta-analysis. Gastroenterology..

[23] Shaukat A, Wang A, Acosta RD, Bruining DH, Chandrasekhara V, Chathadi KV, Eloubeidi MA, Fanelli RD, Faulx AL, Fonkalsrud L, Gurudu SR, Kelsey LR, Khashab MA, Kothari S, Lightdale JR, Muthusamy VR, Pasha SF, Saltzman JR, Yang J, Cash BD, JM DW, ASGE Standards of Practice Committee (2015). The role of endoscopy in dyspepsia. Gastrointest Endosc.

[24] Moayyedi P, Talley NJ, Fennerty MB, Vakil N (2006). Can the clinical history distinguish between organic and functional dyspepsia?. JAMA..

[25] Taye M, Kassa E, Mengesha B, Gemechu T, Tsega E (2004). Upper gastrointestinal endoscopy: a review of 10,000 cases. Ethiop Med J.

[26] Odeghe EA, Adeniyi OF, Oyeleke GK, Keshinro SO (2019). Use of alarm features in predicting significant endoscopic findings in Nigerian patients with dyspepsia. Pan Afr Med J.

[27] Ebigbo A, Karstensen JG, Bhat P, Ijoma U, Osuagwu C, Desalegn H, Oyeleke GK, Gebru RB, Guy C, Antonelli G, Vilmann P, Aabakken L, Hassan C (2020). Impact of the COVID-19 pandemic on gastrointestinal endoscopy in Africa. Endosc Int Open..

[28] Obayo S, Muzoora C, Ocama P, Cooney MM, Wilson T, Probert CS (2015). Upper gastrointestinal diseases in patients for endoscopy in South-Western Uganda. Afr Health Sci.

[29] Kayamba V, Mulenga C, Mubbunu M, Kazhila L, Hodges P, Kelly P (2022). Association between oesophageal cancer and biomass smoke exposure: a case-control study. Ecancermedicalscience..

[30] Kayamba V, Zyambo K, Mulenga C, Mwakamui S, Tembo MJ, Shibemba A, Heimburger DC, Atadzhanov M, Kelly P (2020). Biomass smoke exposure is associated with gastric Cancer and probably mediated via oxidative stress and DNA damage: a case-control study. JCO Glob Oncol..

[31] Kayamba V, Bateman AC, Asombang AW, Shibemba A, Zyambo K, Banda T, Soko R, Kelly P (2015). HIV infection and domestic smoke exposure, but not human papillomavirus, are risk factors for esophageal squamous cell carcinoma in Zambia: a case-control study. Cancer Med.

[32] Allum WH, Blazeby JM, Griffin SM, Cunningham D, Jankowski JA, Wong R (2011). Association of upper gastrointestinal surgeons of Great Britain and Ireland, the British Society of Gastroenterology and the British Association of Surgical Oncology. Guidelines for the management of oesophageal and gastric cancer. Gut..

[33] Ray-Offor E, Obiorah CC (2021). Topography and morphology of gastric Cancer in Nigeria: a dual institution review of 622 upper gastrointestinal endoscopies. Cureus..

[34] Galbán E, Arús E, Periles U (2012). Endoscopic findings and associated risk factors in primary health care settings in Havana. Cuba MEDICC Rev.

